# Ethical dilemmas and reflexivity in qualitative research

**DOI:** 10.1007/s40037-018-0412-2

**Published:** 2018-03-13

**Authors:** Anne-Marie Reid, Jeremy M. Brown, Julie M. Smith, Alexandra C. Cope, Susan Jamieson

**Affiliations:** 1 Leeds Institute of Medical Education, University of Leeds, Leeds, UK; 2 Postgraduate Medical Institute, Faculty of Health and Social Care, Edge Hill University, Ormskirk, Lancashire, UK; 3 Nuffield Orthopaedic Centre, Oxford, UK; 4 Frimley Health NHS Foundation Trust, Surrey, UK; 5 School of Medicine, Dentistry & Nursing, Glasgow University, Glasgow, UK

**Keywords:** Practitioner research, Ethical dilemmas, Reflexivity, Medical education

## Abstract

**Context:**

For medical education researchers, a key concern may be the practicalities of gaining ethical approval where this is a national or local requirement. However, in qualitative studies, where the dynamics of human interaction pervade, ethical considerations are an ongoing process which continues long after approval has been granted. Responding to ethical dilemmas arising ‘in the moment’ requires a reflexive approach whereby the researcher questions his/her own motivations, assumptions and interests. Drawing on empirical studies and their experiences in academic and clinical research practice, the authors share their reflections on adhering to ethical principles throughout the research process to illustrate the complexities and nuances involved.

**Objectives and findings:**

These reflections offer critical insights into dilemmas arising in view of the ethical principles driving good conduct, and through domains which distinguish between procedural ethics, situational ethics, ethical relationships and ethical issues in exiting the study. The accounts consider integrity and altruism in research, gatekeeping and negotiating access, consent and confidentiality, power dynamics and role conflict, and challenges in dissemination of findings. The experiences are based on a range of examples of research in a UK context from managing difficult conversations in the classroom to video-ethnography in the operating theatre.

**Discussion and conclusions:**

These critical reflections make visible the challenges encountered and decisions that must be taken in the moment and on reflection after the event. Through sharing our experiences and debating the decisions we made, we offer insights into reflexivity in qualitative research which will be of value to others.

A Qualitative Space highlights research approaches that push readers and scholars deeper into qualitative methods and methodologies. Contributors to *A Qualitative Space *may: advance new ideas about qualitative methodologies, methods, and/or techniques; debate current and historical trends in qualitative research; craft and share nuanced reflections on how data collection methods should be revised or modified; reflect on the epistemological bases of qualitative research; or argue that some qualitative practices should end. Share your thoughts on Twitter using the hashtag: #aqualspace

## Introduction and context

The importance of procedural ethics, gaining initial approvals for research studies, is well-established and guided by the principles enshrined in the Declaration of Helsinki and the Nuremberg code [1]. Ethical approval procedures are often viewed as a ‘hurdle’ to be surmounted, and arguably overshadow full consideration of the challenges of process ethics, the ethical tensions and dilemmas which arise throughout the practice of research. This paper aims to bring insights into ethical dilemmas which permeate research at all stages, from initial approval through data collection, dissemination of the findings and exiting the study [2]. These insights, developed from the authors’ personal accounts of their own practices, are derived from a range of experiences of medical education research in different settings.

Procedural ethics in medical education is guided by the codes of practice devised by education research bodies such as the British Educational Research Association (BERA) and the American Educational Research Association (AERA). These codes are applied by ethics committees who provide scrutiny at a national or local level depending on the context and nature of the research. For example, in the Netherlands, a national framework to review medical education research has been developed by the Ethical Review Board of the Dutch Association for Medical Education (NVMO) [3]. In the UK, ethical scrutiny is conducted under the auspices of university ethics committees, except where there is patient involvement, in which case clinical ethics committees are involved.

Until relatively recently, medical education research has been given something of a rough ride by ethics committees more used to considering clinical interventional research [4], although such committees are now becoming more familiar with educational research and the ethical issues arising. Guidelines for ethical behaviour are based on the philosophical principles of *beneficence*, do good; *non-maleficence, *do no harm; *respect for autonomy, *self-determination; and *equity, *treat fairly [5]. These principles are intended to guide thinking in applying for ethical approval by encouraging researchers to anticipate the ethical tensions and dilemmas which might arise during the study.

## Guiding principles

The formulation of ethical research principles is based on the deontological approach proposed by Kant [6], whereby moral duty should be guided by principles which transcend circumstances. The utility of these principles in guiding practice is subject to critique on the basis that they may encourage following the ‘letter’ rather than the ‘spirit’ of the principle, or may in fact be too restrictive, limiting the scope and value of the research [7]. In addition, the consequences of research outputs are not entirely within the control of the researcher, and so the principle of non-maleficence, do no harm, may be difficult to achieve in all cases [8].

Guillemin and Gillam [9] distinguish between the domains of procedural ethics (gaining approval) and that of process ethics (ethics in the course of practice). In doing so they recognize that ethics are essentially situational, and that ethically sensitive issues occur ‘in the moment’ as research unfolds [7, 9]. Tracy [2] elaborates further in proposing four domains to guide thinking; *procedural ethics *(approval processes), *situational ethics *(the research context), *ethical relationships *(dynamics between the researcher and participants) and *ethical issues in exiting the study *(completion and disseminating findings). This approach recognizes the ongoing, unanticipated challenges which might arise beyond gaining approval for the study and which require a high degree of researcher ‘reflexivity’ in responding ethically.

Shacklock and Smyth [10] describe reflexivity as the conscious revelation of the underlying beliefs and values held by the researcher in selecting and justifying their methodological approach. From an epistemological perspective, a reflexive approach recognizes knowledge as constructivist, developed throughout the research process and contingent upon existing understandings and beliefs [11]. Transparency about the researcher’s position and potential biases and assumptions is vital in judging accounts of qualitative research and the authenticity of the findings. This paper seeks to illustrate reflexivity in managing ethical tensions and dilemmas through accounts of ‘practitioner-researchers’, that is, those with a role in academic or clinical practice who also conduct research.

This researcher position is one with a working knowledge of the field of study, a shared identity with participants, sensitivity to competing priorities and as one known to participants outside of the researcher role. This practitioner-researcher position is valuable in developing practice insights. However, it may equally bring assumptions and biases which have ethical implications but which are not commonly made explicit in medical education research. The authors seek to address this by offering critical insights into their personal ethical dilemmas drawing on the ethical principles proposed by Beauchamp and Childress [5], and with reference to the framework proposed by Tracy [2]. As stated, the latter concerns *procedural ethics* (formal approval procedures), *situational ethics* (issues arising specific to context), *relational ethics* (awareness of researcher actions on others) and *exiting ethics* (considerations arising beyond data collection). The authors’ accounts (indicated by their initials) include gatekeeping and negotiating access in procedural ethics, debated by JB and SJ; questions of consent and confidentiality in situational ethics experienced by AC; power dynamics and role conflict in ethical relationships debated by AMR and JS and issues of confidentiality and anonymity which challenged AC in dissemination of her findings.

### Procedural ethics

Procedural ethics, the formal approvals required for a study to commence, are dependent on the justification for the study and a stated commitment to adherence to ethical principles. Even before embarking on the formal application, the good intentions of the research need to be carefully weighed up against the vested interests of the researcher involved. Tracy [2] advocates that researchers practise (self-) reflexivity ‘even before stepping into the field … [to assess] … their own biases and motivations’ (P. 842). An illustration of this is provided by JB, an experienced academic and researcher in postgraduate medical education. He reflects on his role conflict as gatekeeper in pursuit of integrity and altruism in research practice. Gatekeepers are those who give access to a research field, or, ‘are able to control or limit researchers’ access to the participants’ [12, P. 452]. Another aspect of the gatekeeper’s dilemma is described by SJ in relation to her role in managing the research of others. This follows JB’s story.

### Integrity and altruism

*JB:* The Universities UK ‘Concordat to Support Research Integrity’ [13] calls for integrity and research excellence throughout the duration of a project. However, one, often hidden complexity, concerns the personal motivation of the researcher, a troubling question for me in leading a research program funded by a postgraduate medical department. Despite beneficent intentions and careful adherence to research governance processes, I retain lingering doubts as to the extent to which hidden coercive influences impact on participant recruitment.

Medical education research is relatively poorly funded and time pressured as many researchers are practitioners with substantial academic and/or clinical roles and responsibilities. As such, these researchers need to reflect upon, and be transparent about, their competing interests [14, 15]. Although this issue has been highlighted for some time in the field, change has been slow, as evidenced by the relatively low number of competing interests disclosed in a survey of medical education related journals [16]. Walsh [17] suggests that medical education researchers may not deliberately fail to disclose, but rather fail to recognize their own competing interests due to the ‘bias blind spot’.

Bearing this in mind, I am conscious of relying heavily on the goodwill of participants with whom I may have relationships before, during and after the study. I believe that the participant should be exercising judgement based on the validity of the study in which they are consenting to participate, rather than on that relationship. Despite the best intentions to contribute to new knowledge, there must be an open and honest realization that research outputs are key benchmarks to measure how effectively researchers are performing. This dilemma was brought home to me while teaching postgraduate students, one of whom posed the question, ‘Can research ever be truly altruistic?’

Initially I was unsure of how to respond, but on reflection, admitted that in my experience, despite endeavouring to be honest and truthful to our participants, there may be a conflict between our personal motivations as researchers and reliance on the goodwill of participants. These reflections are unlikely to lead to any dramatic changes in practice; however, recognizing this potential conflict can at least remind us never to take for granted the commitment of willing participants.

### Protection versus paternalism

*SJ: *Balancing the aims of beneficence, the value of research to the medical education community, with the principle of non-maleficence, avoiding harm, is an ethical conundrum which I have frequently encountered in managing an undergraduate medical education program. As a member of the medical education research community I am keen to support research, but am conflicted when faced with frequent requests to include our students as study participants. This particularly arises with survey research which is often the subject of such requests. The issue of survey fatigue is well-recognized [18], so much so, that my institution instigated a policy to restrict this [19]. The policy aims to prevent survey fatigue and ensure that students remain well-disposed to completing important internal and external surveys including the National Student Survey [20], the results of which are important key performance indicators for the institution.

On becoming director of a masters’ program in health professions education, tensions arising from my role as gatekeeper were exacerbated. The needs of the postgraduate students to recruit study participants potentially conflicted with the interests of the undergraduates. The availability of undergraduates as study material for postgraduate students and career academics is not new, and two decades ago undergraduate psychology students were described as ‘a captive population with little power’ [21 P. 74]. In a similar vein, Keune et al. [22] noted ethical issues arising in a scenario whereby a surgical resident introduced a mandatory team/trauma simulation training session. There was no indication that this session was also the basis of a research study until the trainees were presented with consent forms on arrival, clearly suggesting possible coercion. Another study explored the motivation of Indian medical students to participate in research conducted in their university and hospital learning environments [23]. Of the 300 participants, 61% admitted to participating against their genuine wishes, of which 26% agreed because a faculty member had asked them and 4% because they had not appreciated the right to refuse.

These examples demonstrate a tension between protecting potential participants and respecting their autonomy to choose whether or not to participate in research. One solution as gatekeeper may be to give agreement for participants to be recruited via a general email to a whole cohort, but prohibit purposive sampling of a specific group who may feel more pressurized. Managing access to students as participants highlights for me the ‘grey area’ between protecting the vulnerable and behaving paternalistically in upholding ethical principles.

## Situational ethics

After formal approval and access has been granted, unanticipated ethical questions may arise due to the specific nature of the research setting [1]. In clinical settings data collection occurs within the course of clinical practice which brings specific challenges. AC, a colorectal surgeon whose doctoral research investigated teaching and learning in the operating theatre grappled with issues of consent in the context of patient autonomy.

### Issues of consent in the operating theatre

*AC: *My research followed principles of naturalistic inquiry [24], capturing the phenomenon of teaching and learning in the operating theatre, as it happened, through ethnographic observation, video and audio-recordings [25]. One challenge of capturing naturalistic data is gaining prior consent of participants, as it can be difficult to predict who may be co-present within an operating theatre. In my study there was potential for inadvertent participants to be captured during data collection, defiling the research principle of autonomy—the right to determine participation or non-participation [5].

One approach to uphold autonomy was in the choice of microphone for audio capture: the ‘XTag RevoMic’ which has a short capture range. This meant that talk at the operating table of consenting participants was recorded but that others entering the operating theatre were not inadvertently recorded. The recording was taken from a camera placed within the operating lamp for open surgery, and from a laparoscopic (internal body) camera so that the site of the operation was captured but not the faces of those present. Assurance of this was key to maintaining trust between myself as researcher and the clinical teams involved.

Potential patient participation in the study also raised issues of autonomy. The study explored interactions between surgeon and trainee, but the patient’s body cavity formed the backdrop for video recordings of hand movements and the interactions involved. This meant that the patient would be co-present during the data collection episode although under anaesthetic. Murphy and Dingwall [26] state that ‘one must distinguish between those for whom the research is likely to be consequential and those who are tangential to it’. The ethics committee deemed that patient participants were peripheral, and that for this non-interventional research, patient consent was not required. General Medical Council (GMC) guidance specifies that doctors may use recordings such as laparoscopic video streaming or images of internal organs for secondary purposes (such as research), without seeking consent from patients, provided that the recordings are captured as part of patient care and are anonymized [27]. In this study recordings were captured by the research team and therefore were not a routine part of patient care.

The clinician participants, in particular the nursing staff, expressed very strong feelings that the patient should also be recognized as having the right to consent in making an informed judgement in whether or not to participate. Despite this presenting an extra hurdle for me as researcher, in my surgeon role I was used to gaining patient consent for operations and agreed that patients should be given the right for their procedure to be part of the study or otherwise. I discussed the study with patients, providing a bespoke ‘plain language’ Participant Information Sheet and consent form. Inevitably this raised separate ethical difficulties as patients were anxious that by consenting, they were agreeing to be operated on by a learner surgeon (given that the study was investigating clinical teaching and learning). I had to deal with patient vulnerability sensitively, explaining that participation in the study or otherwise would have no bearing upon their clinical treatment, or the person performing the operation, emphasizing that this was a non-interventional study. In this way the autonomy of patients to choose to participate was respected. No patient declined to be part of the study on this basis.

## Ethical relationships

The issues arising described by AC highlight the complex nature of ethical relationships in research. The researcher-participant dyad is dependent upon any existing relationship with potential participants and a reassessment of the status of this in the context of the study aims and demands [28]. Managing power dynamics, role conflict and role boundaries in relationships are explored here firstly by AMR and then by JS, each of whom experienced such issues in their respective doctoral studies.

### Power dynamics and role conflict

*AMR: *Power asymmetry is a feature of research with the balance generally considered to be in favour of the researcher who directs the process while the participant responds [28, 29]. Ben-Ari and Enosh [30] argue that this power is actually co-constructed through the process, as participants exert power in shaping knowledge through choosing what to reveal. An illustration of such power dynamics occurred during my study on partnership working in the development and design of a new healthcare degree, commissioned by the Health Authority [31]. As program leader, I led the development and delivery of the curriculum and managed the team involved. This brought me the benefit of access to the university team and senior healthcare managers as participants, but also role conflict in managing relationships as the study progressed.

I was acutely aware of my potential biases and power dynamics as both researcher and manager; when interviewing members of my own team I was sensitive to the ‘ethics of care’ [1]. A specific dilemma arose during an interview with a lecturer when I asked a question from the schedule. Rather than respond to this, the interviewee took the opportunity to air personal grievances. The interview may be viewed as a dialogic process, and my sense of control of the dialogue was challenged by the participant taking ‘counter-control’ [11] in deviating from the schedule. I was unsure of how to react, feeling it inappropriate as researcher to challenge or make a direct response. In order to avoid distractions and regain control, I steered the conversation back to the interview schedule.

Although in the moment I felt that I was acting ethically in prioritizing the quality of the research [32], the issue troubled me later. It seemed inappropriate to raise the grievance with the interviewee in my management role after the event as I had assured participants of the confidentiality of the proceedings. With hindsight, given the power asymmetry in the manager-employee relationship, I now believe that seeking a later opportunity to discuss the grievances would have respected the autonomy of the staff member and provided duty of care. On reflection, a different interviewer may have avoided such circumstances occurring, but in this instance I was expected to conduct my own interview as part of the doctoral training.

### Managing multiple identities in an acute setting

*JS:* My doctoral study involved conducting a multi-site, observational, longitudinal research study, focusing on clinical reasoning development in final year medical students as they transitioned to junior doctors^1^. This required managing a number of boundary issues through my overlapping roles of clinician, researcher, teacher and mentor. As I observed participants in both simulated and clinical workplaces, the ethical challenges of these roles developed. I had to ensure that participants could opt in or out of various clinical scenarios as well as at different data collection points. I also had to be mindful of my own potential biases and the need to treat them equitably, neither advantaging nor disadvantaging them as participants in the study. Furthermore, I had to consider the secondary participants who were the patients and ward staff in each workplace. Given the opportunistic and serendipitous nature of the workplace, audio-recorded consent was initially sought in the moment from secondary participants, followed by full written consent.

My ontological position, in particular my predominantly insider role, required a high degree of self-reflexivity [2]. Despite extensive prior approvals and permissions, I anticipated and faced ethical dilemmas arising from my relationships as both an ‘insider’ (a middle grade clinician), and an ‘outsider’ (clinical settings beyond my own workplace and field of expertise). The potential for role conflict also arose between my clinical and researcher role. It was agreed that should an emergency occur within the workplace, I would intervene if necessary in my clinical role in accordance with the GMC’s Good Medical Practice [33]. Furthermore, I would identify a senior member of staff in advance for each shift to whom I could report any instances of unsafe medical practice. Participants were made aware of my role and ethical position as this may have influenced their decision to be involved.

Ethical dilemmas arising during the study included critical incidents ranging from observing unsafe medical practice to observing primary participants being expected to deal with situations beyond their capabilities. One example occurred in observing a junior doctor who was covering obstetrics, an area in which they had little expertise. The junior doctor had been ‘emergency-paged’ to review an obstetric patient following a significant bleed, where no obstetrician was available. Walking to the emergency, as I unpicked the participant’s clinical reasoning, it became clear that this individual lacked the experiential knowledge and practical skills required. I felt very concerned by this and decided in the moment, that in the interests of non-maleficence (avoiding harm), I would switch role and take an active part in the patient management. Fortunately, shortly after our arrival at the emergency, an appropriate senior doctor appeared and no intervention on my part was required. Had there been unsafe practice, I would have reported the incident following the protocols in place to protect patient safety. Although in this case the situation was resolved, recognising the vulnerability of the junior doctor, I alerted the clinical supervisor to the incident to provide support and ensure that the clinical team debriefed after the event.

## Exiting the study

Ethical dilemmas may continue beyond the study as exemplified by AC, who faced challenging issues in maintaining confidentiality and anonymity in dissemination of her findings which included video and audio data.

### Challenges in dissemination of findings

*AC:* Observational field notes and interview transcripts from my study could be presented at academic meetings in anonymized format. However, data from synchronized video and audio, although names were removed, could not be fully anonymized as voices, gestures and body language rendered individuals identifiable. As a clinical researcher, I was able to select clips and the appropriate mode of presentation for particular audiences. For example, at surgical academic meetings where there was a high chance of identification of participants, which might make them vulnerable, I used subtitled video clips to illustrate research findings. This meant that intonation, pitch and rise and fall of delivery within speech were lost; however, the identity of participants was concealed.

For presentation at medical education meetings there was a lower chance of participant identification. The audience was particularly interested in analysis of exact timing of ‘teaching talk’, the guidance given and subsequent responses of the trainee surgeon including pauses, hesitations and hand movement. To enable this, with support I digitally altered audio data so that the pitch of speakers’ voices was modified to conceal identity. On occasion, specific clips with names removed but not completely anonymized format were presented using speakers’ own voices. Express written permission was received from participants for use of that particular clip, recognizing my ability as practitioner-researcher to gauge the likely sensitivity of the data and the audience. For publication purposes, Jeffersonian [34] transcription notation was used. This technique records pauses, intonation, pace and stresses in the delivery of speech so that the transcript indicates not only what is said, but also how it is said. These different methods of presenting the data were selected in the spirit of non-maleficence, avoiding potential harm to participants, acknowledging that complete anonymity could not be entirely guaranteed. This was made clear to participants at the outset.

## Discussion and conclusion

Qualitative research, by its nature, involves immersion in situations and relationships which are complex and unpredictable. These personal accounts have explored the nuanced nature of ethical tensions and personal dilemmas which have emerged for us, as practitioner-researchers beyond the approval process and arising throughout the research. Although some issues are particular to specific situations, there are common features in the challenge of thinking and acting ethically as a qualitative researcher. These include striving to maintain integrity and altruism, upholding autonomy in gaining consent and access, balancing protection of vulnerable participants with paternalism, managing multiple roles and power relations and avoiding harm in dissemination of findings.

The common thread running through our experiences is the sometimes troubling questions raised which may be difficult to foresee, and even when anticipated, require a response ‘in the moment’ [9], which has ethical consequences. Although the risks involved in JS’s experience of managing an obstetric situation as a clinician-researcher on the wards seem high, and the consequences are immediate, JB’s classroom conversations regarding integrity and altruism in research may have far reaching consequences. Training those learning the craft of research carries significant responsibility as it is key to how future researchers understand ethical principles and manage their own conduct in applying them.

The principles of beneficence, non-maleficence, justice and equity should guide action, but the balancing of these principles effectively from the initial approval through to completion of the study and beyond requires a truly reflexive approach. Through sharing our reflections and insights we hope to raise awareness, not only of the challenges of conducting qualitative research ethically, but also of its value when conducted in a rigorous, ethically informed, thoughtful and reflexive manner. This has implications for those undertaking qualitative enquiry as well the gatekeepers who manage access and for those who prepare and train the researcher of the future.

## Footnotes

1 Smith, JM. The Transition from Final Year Medical Student to Foundation Doctor: The Clinical Reasoning Journey. Dundee; 2015 (unpublished thesis)
